# Report of WHO-sponsored trial of MonA and PotLab colorimeters

**DOI:** 10.1155/S1463924679000626

**Published:** 1979

**Authors:** J. M. Rideout

**Affiliations:** Division of Clinical Chemsitry Clinical Research Centre Harrow Middlesex HA1 3UJ UK


volts, normal operation being from a 9 V internal battery with the same economical 'press to read' power 'on' button. The effect of non-linearity of the calibration scale is minimised by its generous length, over 255 mm, and the expansion of the scale divisions in the more critical lower range. Zero adjustment is by mechanical microadjustment which moves the reference cell from the side of the LED.

REFERENCE

[1] Hjelm, M., Brown S.S. and Mitchell, F.L. *The Journal of Automatic Chemistry* (1979), 1, 214.

### Report of WHO-sponsored trial of MonA and PotLab colorimeters

By J.M. Rideout,
*Division of Clinical Chemsitry, Clinical Research Centre, Harrow, Middlesex HA1 3UJ, UK.*

### Background

The objects of the trial were to field-test, in a variety of climatic conditions, two designs of solid state colorimeter calibrated directly in grams per dl haemoglobin and using a haemoglobinocyanide method for total haemoglobin in blood (based on the recommendations of the International Committee for Standardisation in Haematology) [1] and the Swizzlestick technique [2].

It was originally intended that the trial would take place in two phases. Three digital MonA (Mark 1) colorimeters and three PotLab colorimeters, with the necessary reagents, standards, controls and protocols would be distributed by WHO, Geneva; two to laboratories in South East Asia, two to the Western Pacific and two to the Eastern Mediterranean.

The recipients of the PotLab colorimeters were to carry out haemoglobin estimations only; the recipients of the MonA colorimeters were to carry out total protein and albumin estimations in addition to haemoglobin. When these evaluations had been carried out and the exercises completed, the instruments were to be returned to the Clinical Research Centre (CRC) for checking. The exercises would then be repeated, but the laboratories which originally had a MonA colorimeter would receive a PotLab and vice versa.

In fact the trial was terminated at the end of the first phase partly because the time scale for the exercise became protracted but primarily because sufficient information was forthcoming to make the second phase unnecessary.

In addition to the WHO-sponsored trials, Medical Research Council staff have carried out field trials of the MonA colorimeter system on five occasions in The Gambia and once in Nepal. Some aspects of the experiences gained in these trials have been combined with the WHO reports to produce this assessment.

Both colorimeters use a green light-emitting diode as the light source, thus obviating the need for a conventional filter. PotLab represents an attempt to design the cheapest possible haemoglobinometer. MonA represents a more sophisticated instrument. The observed shortcomings of each of the instruments are as follows:

### PotLab

(1) The 'balance' indicators, small red light-emitting diodes (LEDs) are difficult to see in conditions of high illumination.

(2) Balance is difficult to achieve. As the point of balance is indicated when one LED goes out and the other comes on, it is easy to 'overshoot'. There is a tendency for operators to spend a lot of time trying to produce a 'no lights on' condition.

(3) The mode of operation of the light source (LED) makes it susceptible to thermal drift.

(4) A combination of (2) and (3) produces non-reproducibility of results.

(5) The instrument needs carefully matched cuvettes.

### MonA

(1) The LED numerical display is difficult to see in conditions of high levels of illumination.

(2) The instrument needs carefully matched cuvettes.

(3) In conditons of high humidity the instrument did not indicate the correct value when the 'high calibrate' button was pressed. With one exception, calibration had returned to normal by the time the colorimeter was checked at the CRC.

(4) In one colorimeter, part of the optical section had been damaged by fungal attack.

All MonA colorimeters returned to the CRC have been fitted with an improved optical section which is less susceptible to fungal attack and less sensitive to cuvette imperfections. Two modified colorimeters are presently undergoing field trials.

The circuit boards of the returned colorimeters have been protected with a silicone rubber coating in an effort to reduce the effects of high humidity. This treatment has only been partly successful and work is in progress to identify the part or parts of the instrument sensitive to humidity.

### General assessment of the usefulness of the system

A colorimeter, a box of cuvettes and the necessary packing material will fit into a cardboard box 21 x 20 x 13 cm; with reagents, etc., the whole can be packed into a box 27 x 33 x 29 cm. Colorimeter systems have been sent by air mail to various parts of the world without suffering damage, and can be used immediately.

The portability of the system has been further demonstrated by the fact that haemoglobin estimations have been performed in native schools and villages, in the absence of a local electricity supply. In addition the accuracy and precision obtained with the MonA colorimeter was previously unobtainable using colour comparison methods.

In many laboratories in developing countries it is impossible to get reproducible readings from colorimeters connected to the local electricity supply because of voltage fluctuations. In these cases the MonA colorimeter has given a significant improvement in precision.

### Conclusions

The WHO/MRC trials have provided a considerable amount of information which indicates that there is a need for two colorimeters with completely different specifications:

(I) A field instrument for use by health care workers in village clinics that can measure haemoglobin in a drop of undiluted blood. A design for a suitable system is being considered.

(II) A general purpose colorimeter for a rural hospital laboratory capable of measuring haemoglobin and at least five other chemical constituents of blood. The instrument, which would be a Mark 2 version of the MonA colorimeter as evaluated in the field trials described above, should incorporate the following features:

(1) It should have two modes of operation, COMPARATIVE and ABSOLUTE.

(a) In the COMPARATIVE mode the following features are required:

(i) A continuously variable scaling factor to allow the operator to set the digital display to the concentration units value of a standard being measured.

(ii) Alteration of the scaling factor control should have minimum effect on the blank (zero) setting of the instrument (ie. 7% for 10:1 range).

(iii) The operator should be able to vary electrically the position of the decimal point. The decimal point should be switchable to one of three positions 9.99, 99.9 or 999.0. A 0.999 position would not be needed in clinical chemistry although it is provided by some instrument manufacturers.

(iv) Leading zero suppression should be incorporated to reduce the possibility of reading errors by the operator. Examples of leading zero suppression:

01.0 with leading zero suppression becomes 1.0
00.1 with leading zero suppression becomes 0.1

(2) Other features

(a) The display should be capable of indicating less than zero so that the operator can check if the instrument has drifted below zero. A numerical indication of the degree of drift is desirable in the prototype Mark 2 design. If the circuit is found to be essentially drift-free it should only be necessary on the production instrument to indicate that some negative drift has occurred.

(b) A liquid crystal display should be used, with three digits of similar size to the Mark 1 display.

(c) The optical section should be isolated within the main case of the instrument to allow the electronic section to be made liquid proof.

(d) The optical design should be a twin beam configuration to allow the operator to eliminate any difference between blank and test cuvette etc.

(e) As with MonA Mark the instrument should be battery powered but with options on the battery configuration to allow for local availability of batteries. A single battery is preferred to a number of batteries serially connected because of contact problems in the tropics. An indication of battery state is desirable.

A colorimeter to this specification has been designed and a prototype constructed at the Clinical Research Centre.

ACKNOWLEDGEMENTS
Reports from several field trials have been included in this assessment and help of the following is gratefully acknowledged:
Mr. C.R.M. Bangham, M.R.C. Fellow, Lincoln College, Oxford.
Mr. A. Boulter, P.O. Box 108, Kathmandu, Nepal.
Dr. E.H. Daoud, National Health Laboratory, P.O. Box 287, Khartoum, Sudan.
Dr. C.S. Lee, P.O. Box 113, Suva, Fiji.
Mr. B.T. Suitters and the staff of the Health Laboratory, Jakarta, Indonesia.
Dr. A. Wilkins and the staff of M.R.C. Laboratories, Fajara, The Gambia.

REFERENCES

[1] WHO Consultation on Standardisation in Haematology, Bilthoven, April/May 1975, Document LAB/75.3.
[2] J.M. Rideout, A. Renshaw and M. Snook. Swizzlestick: a novel positive displacement microliter diluting device. *Analytical Biochemistry* (1978) 19, 747-751.

Specialist Periodical Reports

**Photochemistry Vol. 9**

Senior Reporter: D. Bryce-Smith, *University of Reading*

This volume is the ninth in the series of reviews in the field of photochemistry and covers the literature published between July 1976 and June 1977.
"The senior reporter and his colleagues are to be congratulated on a demanding assignment in which a very high standard of performance has been set and consistently achieved." – *David I. Schuster, Journal of Medicinal Chemistry, reviewing Vol. 8*

Hardcover 640pp 8¾" x 5⅝" 0 85186 085 0 £38.00 ($83.50)

**Brief Contents:**
**Part 1: Physical Aspects of Photochemistry**
Spectroscopic and Theoretical Aspects; Photophysical Processes in Condensed Phases; Gas-phase Photoprocesses.
**Part 2: Photochemistry of Inorganic and Organometallic Compounds**
**Part 3: Organic Aspects of Photochemistry**
Photolysis of Carbonyl Compounds; Enone Cycloadditions and Rearrangements – Photoreactions of Cyclohexadienones and Quinones; Photochemistry of Olefins, Acetylenes, and Related Compounds; Photochemistry of Aromatic Compounds; Photoreduction and oxidation; Photoreactions of Compounds containing Heteroatoms other than Oxygen; Photoelimination.
**Part 4: Polymer Photochemistry**
**Part 5: Photochemical Aspects of Solar Energy Conversion**

**Spectroscopic Properties of Inorganic and Organometallic Compounds Vol. 11**

Senior Reporter: E.A.V. Ebsworth, FRSE, *University of Edinburgh*

The eleventh volume in this highly successful series covers the literature published during 1977.
"I have found Volume 8, as I have found the previous volumes, an invaluable, informative reference source. The high standards established by previous volumes are maintained and Volume 8 continues to serve the primary function for which the series was designed. Books that no reference library should be without." – *Thomas G. Attig, Journal of the American Chemical Society, reviewing Vol. 8*

**Brief Contents:**
Nuclear Magnetic Resonance Spectroscopy; Nuclear Quadrupole Resonance Spectroscopy; Microwave Spectroscopy; Vibrational Spectra of Small Symmetric Species and of Single Crystals; Characteristic Vibrational Frequencies of Compounds Containing Main-group Elements; Vibrational Spectra of Transition-element Compounds; Vibrational Spectra of some Co-ordinated Ligands; Mossbauer Spectroscopy.

Hardcover approx 453pp 8¾" x 5⅝" 0 85186 103 2 £32.00 ($70.50)

Orders should be sent to your local bookshop or: The Chemical Society, Distribution Centre, Blackhorse Road, Letchworth, Herts SG6 1HN.

**The Chemical Society**

